# A Survey on Proof of Sequential Work: Development, Security Analysis, and Application Prospects

**DOI:** 10.3390/e28010033

**Published:** 2025-12-26

**Authors:** Jingjing Zhang, Yinxia Ran, Xiuju Huang, Cong Zuo, Junke Duan, Yun Pan, Licheng Wang, Jingtao Wang

**Affiliations:** 1 School of Computer and Cyber Sciences, Communication University of China, Dingfuzhuang East Street, Beijing 100024, Chinaranyinxia@163.com (Y.R.); jingtao0621@163.com (J.W.); 2 School of Mathematics and Computer Science, Longnan Normal University, Longnan 742500, China; 3 School of Cyberspace Science and Technolog, Beijing Institute of Technology, Beijing 100081, China; 4 Shandong Raone Intelligent Technology Co., Ltd., Jinan 250013, China

**Keywords:** timed cryptography, time-lock puzzles, proof of sequential work, verifiable delay functions

## Abstract

Proof of sequential work (PoSW), as an emerging cryptographic primitive, is designed to provide a verifiable method for proving that a computational process has incurred a real and continuous expenditure of time. This characteristic demonstrates its significant application potential in decentralized systems, time-stamping services, and trusted computing. This paper systematically reviews and discusses the developmental trajectory, typical variants, potential attacks, and diverse applications of PoSW. Concurrently, it places a special emphasis on analyzing the evolutionary path and application scenarios of its important special case—the verifiable delay function (VDF) aiming to provide a comprehensive reference for research and practice in related fields.

## 1. Introduction

Proof of Sequential Work (PoSW), as a core branch of timed cryptography, introduces the temporal dimension into security models to construct cryptographic primitives with enforced sequential execution properties. It holds significant application value in the blockchain domain, and its development was motivated by the need to address certain inherent limitations of traditional Proof of Work (PoW) mechanisms. Its theoretical origins can be traced back to Timothy May’s 1993 concept of “sending information into the future” [1], while the true theoretical foundation was established by Rivest, Shamir, and Wagner in their seminal 1996 paper [2]. This research not only systematically articulated the theoretical framework of Time-Lock Puzzles (TLP) and Timed-Release Cryptography (TRC) for the first time but, more importantly, established a methodology for time-controlled encryption based on computational complexity (rather than trusted third parties), charting the course for subsequent development of delayed cryptographic primitives like PoSW.

**Figure 1:**
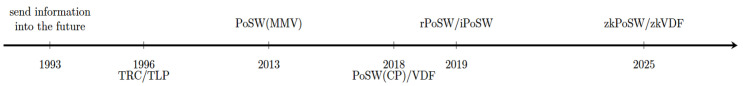
Delayed ideas development timeline.

With the deepening of theoretical research, the PoSW system has evolved from basic concepts to a mature theoretical framework. The PoSW proposed in 2013 [3] overcame the limitations of TLP in public verifiability, while the formally defined Verifiable Delay Function (VDF) in 2018 [4] further strengthened the uniqueness property of solutions, forming a complete theoretical spectrum. These core breakthroughs have been continuously refined by numerous scholars, with related research [3,5,6,7,8,9,10,11,12,13,14,15,16] collectively building the theoretical system of PoSW, whose developmental trajectory is shown in Figure 1. It is particularly noteworthy that TLP, PoSW, and VDF form a closely connected technological progression: TLP serves as the basic PoSW lacking public verifiability, while VDF can be viewed as a special variant of PoSW possessing output uniqueness [4]. This theoretical evolution reflects the continuous development of timed cryptography from single-function to multi-dimensional characteristics.

At the security analysis level, the development of PoSW has maintained a dynamic balance with cryptographic attack techniques. Algebraic VDF constructions represented by MinRoot [4,17] were once considered inherently sequential, but parallel cryptanalysis algorithms based on the “smooth number” strategy [18] theoretically revealed their potential vulnerabilities. Such offensive-defensive interactions have not only promoted the application of more secure fundamental components like iterative hash functions but have also facilitated the continuous improvement of PoSW security models. Meanwhile, the advancement of quantum computing poses severe challenges to number-theoretic-problem-based PoSW, prompting research on post-quantum PoSW [19,20,21] to become an important development direction, providing theoretical foundations for timed cryptography in the post-quantum era.

From an application perspective, PoSW and its derived technologies have achieved the crucial leap from theoretical construction to practical validation. VDF, leveraging its unique non-parallelizable and verifiable properties, has demonstrated significant potential in critical fields such as random number generation [22,23] and blockchain consensus mechanisms. These practical applications not only validate the practical value of PoSW theory but also drive technical optimization through scenario-specific demands, forming a virtuous cycle where theoretical research and application innovation mutually reinforce each other.

The chapters of this article are arranged as follows: Section 2 introduces the developmental history of PoSW; Section 3 mainly discusses whether PoSW can be uniquely verified; Section 4 primarily discusses the post-quantum developments of PoSW; Section 5 analyzes the types of attacks on PoSW; Section 6 mainly describes the application of PoSW; Section 7, summary.

## 2. The Development of Proofs of Sequential Work

The evolution from traditional Proof of Work (PoW) [24] to Proof of Sequential Work (PoSW) [3] stems from a core motivation to redefine the verification dimension of computational puzzles. PoW mechanisms, epitomized by Bitcoin, are fundamentally predicated on highly parallelizable computations. This characteristic dictates that computational advantage is directly proportional to the scale of hardware accumulation, resulting in a linear—or even super-linear—correlation between capital investment and rewards. This dynamic not only instigates an unbounded energy arms race but also exacerbates the trend toward computational centralization.To circumvent this dilemma, PoSW shifts the definition of the scarce resource from “spatial parallel computation” to “temporal sequential execution.”

**Figure 2:**
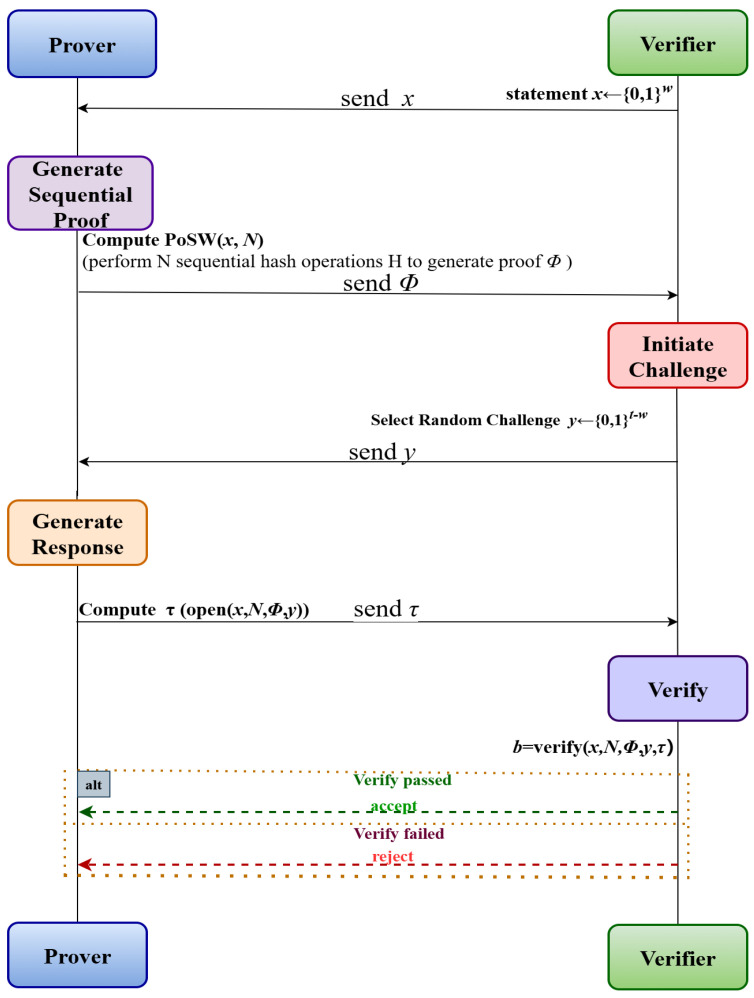
Flowchart of the PoSW algorithm.

Currently, PoSW is increasingly established as a critical cryptographic primitive, widely utilized in constructing VDF and hybrid consensus mechanisms to assist Proof of Stake (PoS) systems in mitigating security challenges such as long-range attacks, rather than serving merely as a standalone mining protocol. The workflow of the PoSW is illustrated in Figure 2 below.

PoSW consists of three algorithms:

- $\mathrm{Setup}(1^{\lambda },N)\to pp$: On input a security parameter λ and sequential work parameter *N*, outputs public parameters pp.
- $\mathrm{Prove}\left(pp,x\right)\to \left(\Phi ,\tau_{\mathrm{ready}}\right)$: On input public parameters pp and a statement $x\in {\left\{0,1\right\}}^{*}$, performs *N* sequential hash operations and outputs a proof Φ and a ready state $\tau_{\mathrm{ready}}$.
- Verify(pp,x,Φ,y,τ)→{0,1}: On input pp, statement *x*, proof Φ, challenge *y*, and response τ, outputs 1 if the proof is accepted, and 0 otherwise.

**Remark** **1.** *The ready state $\tau_{\mathrm{ready}}$ output by Prove contains the necessary information for the prover to subsequently generate the response τ upon receiving the challenge y. The specific derivation of τ from $\tau_{\mathrm{ready}}$ and y is deterministic and is implicitly defined within the verification logic.*

In addition to PoSW, time-delay cryptographic primitives such as TLP and VDF share the core concept of proving the passage of time by enforcing inherently non-parallelizable sequential computation. Table 1 provides a detailed comparison of the three in terms of definitions, functions, construction foundations, key features, typical applications, and time and space complexities, clearly highlighting their distinctions.

As an early exploration in this field, PoSW aims to enable a prover to generate a succinct proof certifying that a certain amount of work has been completed. In 2013, Mahmoody, Moran, and Vadhan (MMV) [3] constructed the first publicly verifiable PoSW scheme using depth-robust directed acyclic graphs. However, this scheme suffered from significant overhead (O(N)) in both time and space, and its proofs lacked uniqueness, leaving a crucial direction for subsequent research.

**Figure 3:**
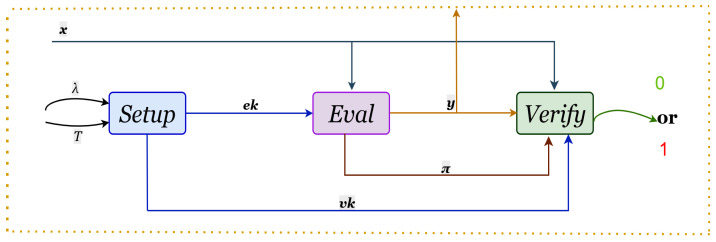
Flowchart of the VDF algorithm.

To address the efficiency issues, Cohen and Pietrzak (CP) proposed an improved PoSW scheme in 2018 [10]. Under the random oracle model, their construction reduced the space complexity to a logarithmic level (O(log(N))), making it one of the most efficient constructions to date. However, the lack of uniqueness remained unresolved. This fundamental shortcoming prompted the academic community to devise new delay primitives with deterministic output. Concurrently, to solve the problem of manipulation resistance in public randomness beacons, Boneh et al. [4] formally defined VDF, which is regarded as a special type of PoSW whose core distinction and key advantage lies in guaranteeing a unique output. The workflow of the VDF is shown in Figure 3 below.

VDF is a combination of three algorithms:

- $\mathrm{Setup}(1^{\lambda },T)\to pp$; returns public parameters pp given a security parameter λ and delay parameter *T*.
- Eval(pp,x)→(y,π); returns y∈Y given x∈X and a proof π.
- Verify(pp,x,y,π)→{0,1}; returns 1 if *y* is the correct output of input *x*.

This definition swiftly catalyzed a surge in constructions, with Pietrzak [7] and Wesolowski [14] contemporaneously proposing two influential VDF schemes based on groups of unknown order. Their efficient verification and concise proofs became cornerstones for future research. Meanwhile, De Feo et al. [6] pioneered a VDF framework based on supersingular isogenies, opening a new direction towards post-quantum security, albeit with challenges related to parameter and storage overhead.

During the burgeoning development of VDFs, the exploration of PoSW also advanced towards ensuring uniqueness and extending functionality. Addressing the uniqueness problem, Aaron Bi Zhang [16] proposed a “Unique Proof of Work” in 2019, which achieved weak uniqueness through statistical methods, providing inspiration for subsequent research. For a more detailed description regarding uniqueness, please refer to Section 3. However, a strictly unique black-box construction remains an open problem. In terms of functional extensions, researchers have combined PoSW with zero-knowledge proofs. When the strict uniqueness of a VDF is not essential, the more efficient zero-knowledge Proof of Sequential Work (zkPoSW) [11] was constructed, demonstrating the flexibility of PoSW.

The exploration of theoretical and security boundaries is a focus shared by both fields. Researchers have proven that constructing compact VDFs with perfect uniqueness in the Random Oracle Model (ROM) is impossible [25,26]. Guan et al. [27] definitively resolved the existence problem of VDFs in the ROM, demonstrating that sequentiality and uniqueness cannot coexist in this model. In the realm of PoSW, in 2021, Chung et al. [12] confirmed through a rigorous re-analysis that the CP [10] scheme remains secure in a quantum computing environment, laying a solid theoretical foundation for the protocol’s long-term application. These theoretical studies have clearly delineated the security guarantees, limitations, and future research directions for both primitives.

**Figure 4:**
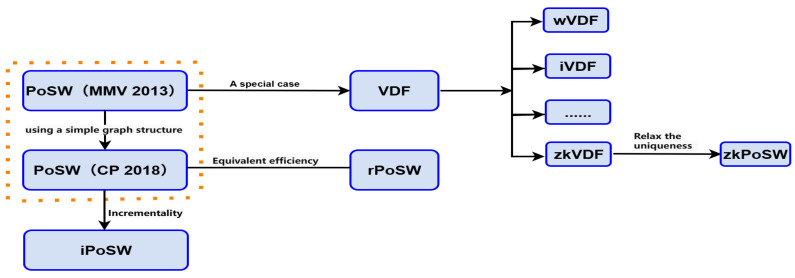
PoSW development process (simplified).

Overall, the development of PoSW and VDFs exhibits an evolutionary trajectory from the general to the specific, from pursuing efficiency to emphasizing uniqueness, and subsequently to a path of mutual inspiration and co-development. A more intuitive flowchart depicting the development trajectory of PoSW is shown in Figure 4. PoSW pioneered the concept of publicly verifiable sequential computation, while VDFs, driven by the pressing needs of applications like randomness beacons, formalized the critical property of uniqueness and realized efficient constructions. Today, the two primitives inspire each other in frontier directions such as incremental computation [5,9], zero-knowledge applications [11], and post-quantum designs [12,20], jointly advancing the theoretical and practical maturity of delay-based cryptography. Challenges remain, such as the construction of a strictly unique PoSW, enhancing the practicality of zero-knowledge VDFs (zkVDFs), and developing more advanced post-quantum schemes.

The main types of PoSW developed so far include the MMV version, CP version, incremental PoSW(iPoSW), reversible PoSW(rPoSW), VDF, and zkPoSW. Table 2 below summarizes the functional and security properties of these delayed cryptographic primitives, along with their underlying construction principles. Prior to comparing the attributes of different PoSW variants, this paper first provides formal definitions for the functional and security properties addressed in Table 2:

- **Sequentiality:** The computation must be executed sequentially; increasing the number of parallel processors cannot significantly shorten the computation time.
- **Public Verifiability:** Any verifier, after obtaining the public parameters, can quickly verify the correctness of the proof without repeating the entire computation.
- **Uniqueness:** For a given input, there exists at most one verifiably acceptable output within the valid proof time.
- **Incrementality:** It allows sequential addition of computation based on an existing proof and generates a new proof covering the extended process without restarting from the beginning.
- **Reversibility:** The computation process can proceed in the reverse direction for verification, meaning it can backtrack from the output to verify the input state.
- **Zero-Knowledge:** The proof can verify that the computation was executed correctly without revealing any information about the computation process beyond the conclusion.
- **Black-Box Construction:** The construction of the scheme relies solely on the functional interface of the primitive, without involving its internal implementation details.
- **Graph-Based Structure:** The security of the scheme depends on the mathematical properties of graph-theoretic structures (e.g., directed acyclic graphs).
- **Number-Theoretic Assumption:** The security of the scheme is based on the hardness assumption of number-theoretic problems.

**Remark** **2** *The following abbreviations are used: Sequentiability (Se), Public Verifiability (PV), Uniqueness (U), Incrementality (I), Reversibility (R), Zero-Knowledge (ZK), Black-Box Construction (BBC), Graph-Based Structure (GBS), and Number-Theoretic Assumption (NTA). An √ indicates that the primitive possesses this characteristic. A × indicates that the primitive does not possess this characteristic or that this aspect was not mentioned in the landmark research papers.*

Despite the emergence of various primitives with similar nomenclature and forms in the cryptographic literature, such as VDF, PoSW, and TLP, in-depth analysis reveals that they fundamentally share a unified objective: addressing the core problem of “how to verifiably demonstrate that a substantial, mandatory sequential time interval has elapsed.” Consequently, the common attributes of these primitives can be abstracted and unified under a general definition: Verifiable Sequential Computation.

Verifiable Sequential Computation is a cryptographic primitive that enables a prover to demonstrate that it has invested a predetermined duration of sequential computation time—which cannot be significantly accelerated through parallelization—to solve a problem. Its core characteristics include:

- **Sequentiality:** The intrinsic structure of the computation ensures that the required time cannot be substantially reduced by increasing the number of parallel processors. This constitutes the most fundamental distinction from traditional PoW.
- **Verifiability:** The process of verifying the correctness of the computation is substantially faster than performing the computation itself, typically requiring time polylogarithmic or constant in the computation time of the prover, thereby ensuring the lightweight nature and scalability of the protocol.
- **Uniqueness:** (Optional, but crucial for VDFs) For a given input, the output is uniquely determined. This functional property enables the result to serve as a trusted random source or timestamp.

The definition of VDF emphasizes that the computation time is fixed regardless of parallelism, while verification is fast [28], with its core being verifiability and delay. The definition of PoSW directly indicates that its goal is to prove that the prover has invested *N* sequential computation steps [10]. The essence of the puzzle-solving process in TLP is a type of work that requires fixed sequential computation to complete [2]. All these primitives aim to provide a reliable proof of elapsed time (PoET), and their application scenarios are built upon consensus on trusted time passage. Therefore, classifying them as verifiable sequential computation is appropriate and highly generalizable.

## 3. Uniqueness of Proof of Sequential Work

In 2013, Mahmoody, Moran, and Vadhan (MMV) constructed the first publicly verifiable PoSW [3]. This protocol ingeniously combines depth-robust directed acyclic graphs, sequential hash functions, and collision-resistant hash functions, requiring a prover (solver) to complete a sequential computational task proportional to the depth of the graph. Subsequent research pointed out that the protocol proposed by MMV, as well as some later works, suffered from the problem of non-unique proofs, meaning that multiple valid proofs could exist for the same computational puzzle. This was identified as an open problem to be addressed [10]. This characteristic poses challenges for applications requiring deterministic outcomes, such as blockchains.

The non-uniqueness of the output in PoSW [3] is an inherent and fundamental flaw that violates the principles of determinism and fairness essential for decentralized consensus [16]. This deficiency can lead to a series of severe negative consequences:

- **Grinding Attack**: The existence of multiple valid proofs allows malicious actors to repeatedly “grind” and select the most advantageous proof, thereby gaining an unfair competitive edge. This attack ultimately results in the centralization of network consensus power [16].
- **Double-Spending**: Grinding attacks further create the potential for double-spending [29]. An attacker can leverage this advantage to privately create a longer chain, invalidating a previously broadcasted transaction, and thus double-spend the same funds.
- **Network Forks**: The non-unique output of the protocol can also directly lead to network forks, which disrupt transaction finality and compromise the stability of the entire network.

Therefore, solving the issue of output uniqueness is of paramount importance. It transforms the protocol from a manipulable “game of choice” back into a fair “computational race,” thereby safeguarding the integrity and security of the blockchain network [16].

To optimize the complexity and efficiency of the MMV scheme, Cohen and Pietrzak (CP) proposed an improved construction in 2018 [10]. By employing a more concise class of specific directed acyclic graphs, the CP scheme not only enhanced the overall efficiency of the protocol but also reduced the prover’s space complexity from O(N) to O(logN). Despite these significant performance improvements, the CP scheme still failed to address the fundamental issue. Like the MMV protocol, it also could not guarantee the uniqueness of the proof, leaving this critical limitation persistent in the development of PoSW.

In the evolutionary course of PoSW, researchers also explored its usability from other dimensions. For instance, the incremental PoSW (iPoSW) proposed by Döttling et al. [9] focused on solving the practical challenges of computational task interruption and resumption. Through its innovative “on-the-fly” generation and parallel recomputation techniques, this scheme achieves fault tolerance and task migration with low memory overhead. Concurrently, Abusalah et al. took a different approach, designing a PoSW based on skip lists and invertible permutations [8], also known as reversible PoSW (rPoSW), whose verification speed far exceeds the proof generation speed.

However, throughout the development of PoSW and its numerous variants, a common limitation persists: the lack of uniqueness. The core of many schemes lies in verifying the “work” rather than the “result.” This inherent mechanism prevents them from producing a deterministic, unique output that can be directly trusted by consensus systems. This deficiency not only restricts the application of PoSW in fields requiring high determinism, such as blockchain, but has also made it a long-standing open challenge in the area [10].

Just as PoSW research was mired in the dilemma of uniqueness, the advent of the VDF brought a glimmer of hope for resolving this issue. VDFs were initially proposed to mitigate the risk of prediction and manipulation of public randomness sources [4]. A VDF is considered a special case of PoSW [4], yet its design is fundamentally different: a VDF must not only prove the passage of a sequential computation time but also ensure that this computation produces a unique and publicly verifiable result.

In 2018, Boneh et al. provided the first rigorous formal definition of a VDF [4], specifying its three core properties: Sequentiality, Uniqueness, and Verifiability. Among these, “Uniqueness” is its most fundamental distinction from traditional PoSW, requiring that for any given input *x*, the VDF function f(x) must produce one and only one deterministic, valid output *y*. This property resolves the non-determinism issue inherent in PoSW.

The uniqueness of VDFs is not merely a theoretical claim but is guaranteed by their underlying mathematical constructions, which ensures the unforgeability and determinism of the results. This characteristic constitutes a key advantage of VDFs, as it guarantees the reliability of their outputs, enabling direct application in critical scenarios such as decentralized random beacons and leader election in consensus mechanisms. Any participant can be confident that the VDF output they are verifying is the sole legitimate result corresponding to that specific input. Even with the subsequent emergence of numerous variants like incremental VDFs [4] and zkVDF [11], they all steadfastly maintain “unique verifiability” as a core security property.

Interestingly, as research has deepened, the concept of PoSW has formed a complementary relationship with the properties of VDFs in new application contexts. ZkPoSW [11] is a prime example. In a context where zkVDF are impractical due to prohibitively large proof sizes, zkPoSW makes a strategic trade-off: it deliberately forgoes the pursuit of output uniqueness, focusing instead on proving in zero-knowledge that the “computational process” itself was honest and time-consuming.

By combining proof of exponentiation (PoE) with a proof of knowledge of discrete logarithm (PoKDL), zkPoSW achieves lightweight, zero-knowledge verification of sequential work [11]. It is not concerned with what the final output is, but only that the prover has indeed invested non-parallelizable computational effort. This design philosophy demonstrates that uniqueness is not a necessity in all scenarios. When an application only requires a transient credential certifying the “passage of time” and has no requirement for the determinism of the result, forgoing uniqueness can lead to greater efficiency and practicality.

In summary, the developmental trajectories of PoSW and VDF collectively reflect the cryptographic community’s theoretical exploration and technical trade-offs surrounding the core attribute of “uniqueness.” As a pioneering paradigm for verifiable sequential computation, PoSW faces application limitations in scenarios requiring deterministic outcomes due to its inherent non-uniqueness. VDF, through rigorous mathematical constructions, overcomes this bottleneck, achieving verifiable deterministic output and thereby providing a reliable foundation for critical applications such as decentralized random beacons and consensus protocols. The emergence of new paradigms like zkPoSW signifies a maturation and expansion of research perspectives. In specific contexts, uniqueness can be treated as a negotiable design dimension, traded for enhanced efficiency and flexibility. These cryptographic primitives complement each other, collectively constructing a rich, layered, and dynamically evolving technological ecosystem for verifiable delay computation.

**Critical Examination:** The developmental trajectory of PoSW and VDF should not be perceived as a linear narrative from “flaw” to “perfect solution,” but rather as a concentrated manifestation of profound technical trade-offs. The pursuit of “uniqueness,” while addressing historical challenges, simultaneously exposes and catalyzes novel contradictions.

- **The Cost of Determinism:** While VDF achieves unique output by introducing algebraic structures, it consequently deviates from the elegant simplicity of PoSW built upon the cryptographically neutral foundation of hash functions. Instead, it relies on more complex mathematical problems that introduce potential centralization risks (e.g., parameter selection, advantages in hardware optimization) and vulnerability to quantum threats.
- **The Escalation of Security Assumptions:** The foundational security basis has evolved from the collision resistance of hash functions to more sophisticated assumptions such as the low-order assumption and knowledge assumptions. Although this progression enables advanced functionalities like determinism and zero-knowledge, it concurrently amplifies the analytical complexity of protocols and introduces potential new attack vectors, thereby tethering system security to more advanced and less battle-tested cryptographic hypotheses.
- **The Paradox of Efficiency:** Gains in “uniqueness" and “verifiability" are frequently offset by increases in computational overhead, proof size, or communication costs. Whether it’s the expensive modular exponentiation in VDF, the substantial proof volume in post-quantum schemes, or the proof generation time sacrificed by zkPoSW, these all underscore the inherent tension among functionality, efficiency, and security.

Consequently, the current state of verifiable delay cryptography reveals a core dilemma: here exists no universal primitive capable of simultaneously optimizing the multiple objectives of parallelization resistance, output determinism, proof succinctness, verification efficiency, quantum security, and deployment simplicity. Every improvement constitutes a targeted trade-off.

## 4. Post-Quantum PoSW

The rapid development of quantum computing is profoundly reshaping the security landscape of modern cryptography. As quantum computers transition from theory to practice, traditional public-key cryptosystems, such as RSA and ECC, face a severe threat of being broken in polynomial time by Shor’s algorithm [30]. This challenge directly undermines the foundations of existing cybersecurity. Against this background, research in post-quantum cryptography (PQC) has become particularly important and urgent. Its objective is to design new cryptographic algorithms capable of resisting attacks from quantum computers. The U.S. National Institute of Standards and Technology (NIST) has initiated a post-quantum cryptography standardization project, driving the research and development of algorithms based on mathematical problems such as lattice-based cryptography and hash-based signatures. Developing quantum-resistant cryptographic technologies is not only a strategic necessity to counter future security threats but also a critical cornerstone for ensuring information security in the digital economy era.

In the context of post-quantum security, the work by Abusalah et al. [8] compares their PoSW construction based on random permutations (a symmetric-key primitive) with other contemporary high-efficiency VDF schemes based on the RSA TLP, thereby highlighting the value of their approach. They note that while these RSA-based VDF schemes offer superior verification efficiency, they are not post-quantum secure. Consequently, for applications requiring post-quantum security, their PoSW construction is “arguably still the best option in a post-quantum setting.” The paper also acknowledges that this advantage may only be temporary, mentioning that research on post-quantum VDFs is ongoing and that the technological landscape may evolve in the future.

Currently, the construction of VDF with quantum resistance has become a major research direction in the field of cryptography [19,20,21]. With the rapid development of quantum computing technology, traditional VDF construction schemes based on number theory problems (such as large integer factorization or discrete logarithms) face the risk of being broken by quantum algorithms. For RSA-like schemes (such as Wesolowski [14] and Pietrzak [7]), which rely on the discrete logarithm problem over groups of unknown order, they can be efficiently broken by Shor’s algorithm [30]. Among isogeny-based schemes, the verification process of De Feo et al.’s scheme [6] relies on bilinear pairings, making it vulnerable to quantum attacks. While Shani’s pairing-free scheme [15] avoids pairing operations, it is only applicable for key exchange on automorphic ring elliptic curves and still has limitations in terms of quantum security. VDFs constructed based on the ROM have been proven to be infeasible [27] and cannot be built from primitive black boxes like one-way functions. Consequently, existing schemes have significant vulnerabilities at the quantum security level. Therefore, researchers are dedicated to exploring new VDF construction methods based on quantum-resistant mathematical problems like lattice-based cryptography and supersingular elliptic curve isogenies. These studies not only need to maintain the core characteristics of a VDF, namely sequential computability and efficient verifiability, but also must ensure their security under the quantum computing model. Breakthroughs in this area will provide reliable fundamental cryptographic primitives for key applications in the future quantum computing era, such as secure timestamps [31] and blockchain consensus.

Current quantum-resistant VDF research has formed three core breakthrough paths by using algorithmic modifications and new mathematical tools. In the direction of pairing-free isogeny-based VDFs, Chavez-Saab et al. [20] combined SNARKs to eliminate dependence on bilinear pairings, achieving post-quantum security, quasi-logarithmic verification efficiency, and no trusted setup. At the same time, the collision-resistant hash property of isogeny paths makes them naturally immune to SIDH/SIKE quantum attacks (Jacquemin et al. [32]). In the area of lattice-based VDFs, Bitansky et al. [33] constructed a VDF with a unique proof based on the learning with errors (LWE) assumption, pioneering a new path for applying lattice-based cryptography to VDF construction.

The construction of post-quantum secure VDF is a significant challenge in cryptography. The first VDF construction based on supersingular elliptic curve isogenies was proposed in 2019 [6]. Its evaluation involved computing images of large-degree isogenies, and verification relied on efficient bilinear pairings. However, this scheme is vulnerable to quantum attacks (as quantum computers can break the discrete logarithm problem it relies on) and requires a time-consuming trusted setup. This highlighted the need for developing truly quantum-secure, trustless VDFs.

To overcome these shortcomings, Chavez-Saab et al. [20] explored the construction of a post-quantum secure VDF based on supersingular isogenies in 2021. They used SNARKs, specifically optimizing them for the arithmetic structure in the isogeny setting to achieve better asymptotic efficiency. They developed a VDF construction scheme based on isogenies that has post-quantum security, quasi-logarithmic verification, and requires no trusted setup. This work also constructed a non-interactive argument for supersingular isogeny paths over $F_{p^{2}}$. Nevertheless, at that time, all quantum-secure VDF constructions (including their own) had not achieved an exponential gap between evaluation and verification under the widely studied post-quantum time assumptions [20]. In the same year, Leroux [34] proposed a verifiable random function (VRF) based on a proof of knowledge of secret isogenies. Although it has an exponential efficiency difference between the prover and verifier, it is not suitable for VDFs because it cannot bind the isogeny computation to a specific input and, as a one-time function, it lacks quantum resistance.

The year 2023 saw progress on multiple technical fronts. Guan Tan et al. [21] designed the quantum-secure VDF scheme ZKBdf based on the zero-knowledge proof system ZKBoo [35]. This scheme achieves non-interactivity through the Fiat-Shamir heuristic [36,37]. Its security can be reduced to ZKBoo in the quantum random oracle model(QROM) [38], and it also requires no trusted setup. ZKBdf not only provides detailed algorithms, security proofs, implementation, and evaluation, but it also extends standard VDF functionality by introducing a prover-secret feature. More groundbreakingly, Decru et al. [19] were the first to propose a weak VDF(wVDF) that achieves quantum resistance without relying on a SNARK. This purely algebraic construction uses the computation of large-degree isogenies between supersingular elliptic curves to generate a delay and uses isogenies between products of two elliptic curves based on Kani’s criterion for efficient verification. This scheme offers theoretically very fast verification, opening a new direction for post-quantum cryptography. However, this wVDF faces two major challenges:

1. Its construction itself has limitations and requires that the input curve has an unknown endomorphism ring.
2. It requires a certain degree of parallelism for the evaluator to gain an advantage.

In 2025, Osadnik et al. [39] made an important breakthrough in the practical research of post-quantum secure VDFs by proposing an innovative scheme called “Papercraft.” As the first VDF implementation constructed entirely based on lattice-based cryptography, this scheme has significant post-quantum security features. The research team, through theoretical innovation in succinct argument systems for lattice bases combined with multi-level engineering optimization techniques, successfully implemented the first lattice-based VDF system that can run efficiently on existing hardware platforms. Experimental data show that the system can verify a 6-minute computation in just 7 s, a significant improvement in verification efficiency. This groundbreaking result not only confirms the theoretical feasibility of lattice-based VDFs but also demonstrates their huge potential in practical applications, laying an important foundation for the engineering application of post-quantum cryptography in distributed systems and other fields.

Current quantum-resistant VDF research has formed three core breakthrough paths: pairing-free isogeny-based constructions, lattice-based schemes, and a purely algebraic wVDF. While these schemes defend against quantum threats like Shor’s algorithm, they also address key bottlenecks such as trusted setup [20], grounding VDFs in a mature post-quantum theoretical framework [33], and verification efficiency [19]. However, significant challenges remain: isogeny-based schemes have not yet achieved the exponential gap between evaluation and verification [20]; lattice-based constructions still need practical efficiency optimization; and the breakthrough wVDF [19] is limited by the requirement for an input curve with an unknown endomorphism ring and its dependence on a certain degree of parallelism for the evaluator to gain an advantage. Future research needs to break through these mathematical constraints, optimize proof sizes, and provide a secure parameter system for standardized applications, all while maintaining the core properties of sequential computation and efficient verification. Based on the content above, Table 3 presents a quantitative comparison between classical VDFs and post-quantum VDFs.

Based on the above discussion, research on post-quantum secure VDF has crystallized into three primary technical pathways, achieving key advancements such as the elimination of trusted setups and improvements in verification efficiency. Nonetheless, the field continues to face core challenges, including the failure to achieve the theoretically optimal gap between evaluation and verification efficiency, performance bottlenecks in practical deployment, and limitations imposed by specific mathematical assumptions. Future research must strive to overcome these theoretical and engineering obstacles while preserving the inherent security properties of VDFs, thereby advancing the practical application and standardization of quantum-resistant VDF technology.

**Evaluation:** The primary advantage of classical delay-based cryptographic primitives lies in their foundation upon well-studied and mature number-theoretic hard problems, which provide a robust theoretical framework. Moreover, some schemes have achieved satisfactory verification efficiency under the classical computational model. However, their fundamental flaw is their vulnerability to quantum attacks; the existence of Shor’s algorithm poses a critical threat of compromise. Additionally, some classical constructions rely on trusted setup, which increases deployment complexity and introduces additional security assumptions.

In contrast, post-quantum delay-based cryptographic primitives, developed to counter quantum threats, are fundamentally designed for future security. Their construction is rooted in mathematical problems such as lattice-based cryptography and supersingular elliptic curve isogenies, which are currently believed to be resistant to quantum computer attacks. In terms of research progress, certain schemes have successfully eliminated the trusted setup bottleneck present in classical approaches and have achieved significant improvements in verification efficiency. Furthermore, novel theoretical pathways, such as purely algebraic constructions independent of SNARKs, have been explored. Nonetheless, these emerging schemes face significant challenges: most have not yet achieved the exponential gap between evaluation time and verification time, which is a core theoretical goal for VDF, thereby diminishing their value as delay primitives. Some schemes are constrained by specific mathematical prerequisites, limiting their generality. Additionally, lattice-based and other schemes still encounter substantial engineering optimization pressures in transitioning from theory to practice.

Consequently, the current research landscape indicates that a solution simultaneously optimized across the four dimensions of post-quantum security, efficiency, generality, and practicality remains elusive. Classical schemes fall short in future security, while post-quantum schemes require further breakthroughs in efficiency theory and engineering practice.

## 5. Attacks on PoSW

As a cryptographic primitive designed to publicly prove the investment of sequential computation, PoSW face complex threats across multiple dimensions, ranging from fundamental mathematics and hardware implementation to system integration. These attack vectors not only challenge its theoretical foundations but also exploit technical and logical vulnerabilities in real-world scenarios. As a crucial special case of PoSW, VDF, due to their stricter requirements for uniqueness and verifiability, not only inherit the general security risks of PoSW but also confront more rigorous and unique security challenges. Table 4 summarizes some typical attack methods and their targets.

For cryptographic systems relying on computation chains such as hash chains, precomputation and time-memory trade-off (TMTO) attacks [40] are well-known cracking methods. Such attacks consist of an offline precomputation phase, which prepares lookup tables similar to rainbow tables, and an online query phase. From the perspective of overall attack logic, this approach poses a severe threat to cryptographic systems dependent on computation chains; similarly, PoSW constructions based on sequential hash chains are also vulnerable to such attacks [50]. Moreover, parallel algorithms like Pollard’s rho [41] can further exploit the birthday paradox to find shortcuts in computation chains [50]. Even cutting-edge post-quantum PoSW schemes are not impenetrable. For instance, the first lattice-based PoSW scheme [13] allowed excessively loose norms for solutions in its protocol design, leading to its successful breach by the parallelization attack proposed by Peikert [51] within logarithmic time. This outcome rendered the scheme unable to meet the sequentiality requirement it promised at the design stage i.e., completing computational steps in a predetermined sequential order rather than achieving results quickly through parallelization thus completely violating the core design philosophy of PoSW [51].

For VDFs, their deeper reliance on the computational complexity of mathematical problems exposes them to more severe algorithmic security challenges. Advances in quantum computing pose a potential threat to current mainstream VDF constructions (e.g., RSA-based schemes [2]), as the integer factorization assumption underlying such schemes could theoretically be compromised by quantum computational approaches such as Shor’s algorithm. Furthermore, some studies have subverted the basic assumption that the core operations (e.g., modular square roots) of algebraic VDFs cannot be accelerated in parallel. The cryptanalytic algorithm based on “smooth numbers” proposed by Biryukov et al. [18] can reduce the computational delay of schemes like MinRoot [17] by several to dozens of times through algebraic shortcuts, and this method is rooted in classical computational number theory [52,53,54,55,56,57]. Subsequently, low-order element attacks and potential structural flaws identified in supersingular isogeny cryptography [42] have also successively posed severe threats to the uniqueness and reliability of VDFs. Low-order element attacks exploit the properties of low-order elements in specific mathematical structures to interfere with the VDF computation process and undermine its uniqueness; meanwhile, potential structural flaws in supersingular isogeny cryptography may enable attackers to exploit structural vulnerabilities to bypass normal computational processes, reduce computational delay, and compromise the reliability of VDFs.

At the hardware acceleration level, attackers targeting both PoSW and VDFs can accelerate core computing units, thereby undermining the fundamental assumption that protocols bind computational steps to physical time. Physical-level attacks pose a particularly prominent threat to dedicated hardware, especially VDF hardware designed for extreme performance. Side-channel attacks such as timing attacks and power analysis do not target the algorithm itself but rather steal secrets by analyzing physical information leaked by devices. This is particularly fatal for Trapdoor VDFs (tVDFs), as attackers may extract the trapdoor key used for fast computation through these channels, thereby completely bypassing the delay function [58]. Paradoxically, VDF hardware circuits optimized for speed are often more sensitive and thus more vulnerable to such physical attacks.

At the protocol and system integration level, the non-uniqueness of PoSW proofs opens the door to “grinding attacks” [16]. In competitive scenarios such as blockchain leader elections, attackers can generate a large number of valid proofs and select the most favorable one to manipulate protocol outcomes. Although the unique design of VDFs can resist such attacks, if the soundness of their proof system is compromised, the possibility of forging proofs to achieve the same goal still exists [16].

Additionally, systems based on PoSW are also at risk of economic attacks such as selfish mining [46]. In terms of system implementation, verification nodes for both primitives may be overwhelmed by processing a large number of invalid proofs, or fall into a denial of service (DoS) state due to algorithmic complexity attacks such as hash collisions [47].

Finally, certain VDF schemes introduce unique system-level vulnerabilities. Many constructions require a trusted setup to generate public parameters (e.g., RSA modulus *N* [48]); if this process is compromised, attackers may retain secret factors, thereby breaking all VDF instances based on these parameters. This fully demonstrates that the security of both general PoSW and specific VDFs is a systems engineering task, and their ultimate integrity depends on the weakest link across all stages, from mathematical theory to physical implementation.

## 6. Applications of PoSW

**Figure 5:**
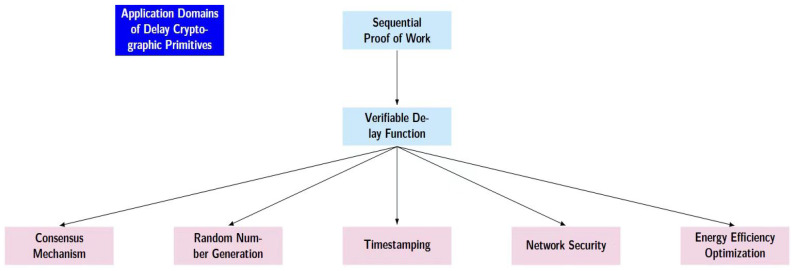
PoSW applications.

In light of social development and progress, the concept of deferred-thought has permeated numerous aspects of our daily lives. This approach, along with related technologies, facilitates the achievement of various objectives across a multitude of domains. Next, as shown in Figure 5, this section will introduce several particularly important application domains.

As an alternative to traditional PoW, PoSW mandates that the computational process be inherently sequential [16]. This paradigm aims to mitigate the substantial waste of computational resources and energy resulting from the parallelizable nature of traditional PoW. Specifically, a PoSW characterized by unique proofs is termed a VDF; this uniqueness property is critical for preventing grinding attacks within blockchain systems. The fundamental advantage of these technologies lies in the fact that participants derive no significant benefit from investing vast resources into parallel computation, thereby circumventing the excessive energy consumption associated with the deployment of large-scale machine clusters typical of Bitcoin mining [16]. For instance, the Chia Network employs a mechanism that integrates PoSW (implemented as a VDF) with “proofs of space”. Since proofs of space require the allocation of disk storage rather than computational time, ordinary users can participate using idle disk space, enabling the system to avoid the high energy consumption inherent in traditional PoW without incurring significant costs [16].

Mirkin et al. [59] proposed *Sprints*, a protocol that integrates Proof of Delay (PoD) with PoW to establish an intermittent mining mechanism. The core of this approach lies in leveraging VDF technology to achieve a balance between energy efficiency and security. While conceptually defined as a distinct primitive, PoD essentially represents an innovative application built upon VDF technology. *Sprints* constructs the initial PoD computation phase by exploiting the “non-parallelizable” and “efficiently verifiable” properties of VDFs, employing the Wesolowski VDF [14] algorithm in its concrete implementation. The mining process is bifurcated into two phases:

- **PoD Phase (Threshold):** This phase constitutes a deterministic process rooted in serial computation. Miners equipped with efficient hardware can complete this task within the allotted time with a 100% success rate, serving as a prerequisite for participation in the subsequent competition.
- **PoW Phase (Competition):** This phase involves traditional probabilistic computation, characterized by a nonce selection process. Due to the inherent stochastic nature of PoW, successful completion of the PoD phase does not guarantee victory.

By utilizing PoD as a preliminary delay and filtering mechanism, this architecture allows for a significant reduction in the duration of the energy-intensive PoW computation (e.g., to merely 5% of the block interval). This approach not only effectively suppresses parallel mining but also significantly mitigates the system’s overall energy consumption and carbon footprint, all while maintaining security guarantees comparable to those of legacy PoW.

In the domains of time verification and execution fairness within distributed systems, delay-based primitives exhibit distinct value. The inherent sequential nature of PoSW facilitates the construction of non-interactive timestamping schemes that are independent of trusted third parties (external services) [3]. This mechanism permits the verification of knowledge concerning documents or data without reliance on centralized services. Building on this foundation, such technologies have evolved further—exemplified by VDF—and are applied within blockchain architectures to enhance fairness. Simultaneously, the characteristic of PoSW requiring a substantial investment of sequential time enables it to function as a universally verifiable CPU benchmark, offering a non-manipulable, objective basis for evaluating computational hardware performance [60].

VDFs, as a special case of PoSW [4,16], play an indispensable role in generating trustless randomness. Conventional randomness generation schemes are susceptible to prediction or manipulation by miners with superior computational power [25,61]. VDFs, through their “time-lock” property, enforce a non-parallelizable computational period before a result is revealed [32,62]. This makes it impossible for any party to accelerate the computation or predict the outcome [16], thereby providing a reliable and manipulation-resistant randomness beacon for critical processes in blockchains, such as leader election and block proposer selection [23]. This feature also significantly enhances the security of commit-reveal schemes by preventing participants from withholding their reveals if the outcome is unfavorable [61].

The scope of application for delay-based cryptographic primitives has been further expanded, providing a solid security foundation for the construction of advanced decentralized applications. In Proof of Stake (PoS) protocols and distributed randomness generation, VDF are utilized to select validators or leaders in a probabilistically fair manner, effectively preventing malicious actors from predicting or manipulating the election process [23]. The application of VDFs is particularly critical in highly sensitive domains such as finance. As noted in the literature, in scenarios requiring the generation of randomness beacons from public sources, such as stock prices, VDFs ensure security by enforcing a sufficient computational delay in beacon generation; this prevents experienced traders equipped with substantial computational power from manipulating market prices for personal gain [32]. Furthermore, researchers have leveraged the properties of VDFs to pioneer trustless Proofs of Retrievability and Reliability (PORR) schemes, ensuring that storage providers genuinely store data replicas rather than generating them on the fly upon challenge. Collectively, these innovations have paved new technical pathways for designing resource-efficient and highly secure blockchain systems [10,31,63].

In summary, time delay cryptographic primitives are fundamentally reshaping the technical paradigms for constructing trusted and efficient decentralized systems, particularly PoSW and VDF. By enforcing mandatory sequential computation, they transform the abstract concept of “trusted time passage” into publicly verifiable digital proofs, thereby addressing inherent flaws of traditional consensus mechanisms across multiple critical dimensions. From replacing energy-intensive PoW and ensuring fairness in distributed random number generation to building trustless timestamping and verifiable storage schemes, the application of these technologies has progressed from theoretical exploration to widespread practice. They establish a solid foundation of both resource efficiency and high security for next-generation blockchains and distributed applications. Their core value lies in cryptographically anchoring and efficiently utilizing key physical world resources—such as time, computation, and storage—without reliance on centralized authority, providing essential technical support for building a fairer, more transparent, and sustainable digital economic infrastructure.

## 7. Conclusions

This paper provides a systematic treatment of timed cryptography. It first delineates the developmental trajectory of PoSW, offering a comparative analysis of three cryptographic primitives—VDF, PoSW, and TLP—to elucidate their technical distinctions and application boundaries. The study then examines the theoretical and practical implications of the uniqueness property for VDF and PoSW, while also assessing current research progress and countermeasures against quantum computing threats for these primitives. Finally, the paper systematically synthesizes predominant attack models targeting contemporary PoSW protocols and their corresponding defense mechanisms, concluding with an outlook on practical application scenarios and future developments.

Promising future research directions include: (1) constructing PoSW with unique output properties, which remains a fundamental theoretical challenge in the field; (2) developing quantum-resistant VDF constructions, representing one of the most pressing and strategically significant research challenges; (3) exploring functional integration mechanisms across different VDF variants—specifically investigating whether modular design approaches can achieve complementary advantages, thereby opening new technical pathways. These directions represent key focal points for advancing timed cryptography, holding critical importance for both theoretical refinement and practical applications of verifiable delay functions.

## Figures and Tables

**Table 1:** A comparative analysis of the core attributes of VDF, PoSW, and TLP, highlighting their differences including computational complexities.

Attribute	VDF [4]	PoSW [3]	TLP [2]
**Academic Definition**	Emphasizes fixed, verifiable, and fast verification of a time-based proof.	Emphasizes a strong sequential computation proof, requiring a fixed number of sequential computational steps to be input and solved.	Emphasizes that encrypted information can only be decrypted after a fixed time.
**Core Function**	Provides a reliable and non-parallelizable proof of time, yielding a unique output.	A proof that a fixed amount of sequential work has been completed; does not necessarily require a unique output.	Binds a secret message to a forced delay, enabling timed release.
**Construction Foundation**	Based on cryptographic assumptions of hardness.	Based on the hash grid (DAG).	Based on cryptographic assumptions of hardness.
**Key Features**	The result is unique and deterministic, and it can be publicly verified.	The proof is for the amount of work done, not necessarily a deterministic result; can be publicly verified.	Only effective for the specific problem being solved; the proof might not be public.
**Typical Applications**	Random beacons, blockchain consensus (PoH), delay encryption.	Timestamps, CPU benchmarks, novel blockchain architectures.	Timed message release, sealed-bid auctions, electronic voting.
**Time Complexity**	**Eval:** Strictly bounded by parallel time *t*. **Verify:** Required to be highly efficient, with a total running time of O(poly(log(t))). *Core:* Exponential gap between evaluation and verification.	**Solving:** An honest solver requires N·poly(n) time. **Verify:** Efficient, requiring poly(n)·polylog(N) time. *Core:* Adversary’s parallel time cannot be significantly less than sequential time (Parallel η-Soundness).	**Solving:** Requires a “precise amount of time” or “pre-determined amount of time”. *Core:* Emphasizes an “intrinsically sequential” nature; computation must be continuous and non-skippable.
**Space Complexity**	Memory size is not explicitly defined, but Eval parallelism is limited to poly(log(t)) processors. Implicitly requires succinct proof size π for rapid verification.	Memory size is not explicitly defined. “Open Questions” note that concrete constructions require Ω(N) storage, identified as a drawback.	Memory size is not explicitly defined. The definition focuses on restricting hardware/parallel resources, mandating that increasing parallel computing power (spatial resources) must not accelerate the solution.

**Table 2:** Properties of different PoSW types.

Type (Reference)	Functionalities			Application Properties			Building Features		
	Se	PV	U	I	R	ZK	BBC	GBS	NTA
PoSW [3]	✓	✓	×	✓	×	×	✓	✓	×
PoSW [10]	✓	✓	×	×	×	×	×	✓	×
iPoSW [9]	✓	✓	×	✓	×	×	×	✓	×
rPoSW [8]	✓	✓	×	×	✓	×	×	✓	×
zkPoSW [11]	✓	✓	×	×	×	✓	✓	×	✓
VDF [4]	✓	✓	✓	×	×	×	✓	×	✓
TLP [2]	✓	×	×	×	×	×	×	×	✓

**Table 3:** Comparison between classic VDF and post-quantum VDF.

Comparison Dimensions	Classic VDF	Post-Quantum VDF
**Theoretical Foundation**	Relies on classical number-theoretic hard problems (e.g., integer factorization, discrete logarithm).	Based on quantum-resistant mathematical hard problems (e.g., lattice, isogeny of supersingular elliptic curves).
**Typical Constructions**	(1) RSA/Unknown Order Group (e.g., Wesolowski [14]; Pietrzak [7]); (2) Bilinear Pairing Group (e.g., De Feo et al. [6]).	(1) Homomorphic + SNARK (e.g., Chavez-Saab et al. [20]); (2) Lattice-Based (e.g., Bitansky et al. [33], Osadnik et al. [39]); (3) Pure Algebraic wVDF (e.g., Decru et al. [19]).
**Quantum Security**	**Insecure**. Core hard problems (e.g., discrete logarithm) can be solved in polynomial time by Shor’s algorithm.	**Designed to be secure**. Based on mathematical problems currently believed to resist quantum computer attacks.
**Core Advances**	Under the classical computing model, achieved rigorous definitions and constructions for sequentiality, uniqueness, and efficient verification.	(1) Elimination of trusted setup; (2) Improved verification efficiency (quasi-logarithmic, sub-second verification); (3) New theoretical approaches (pure algebraic constructions independent of SNARKs).
**Challenges**	(1) **Quantum Attacks**: Fundamental threat of being broken by quantum computers; (2) **Trusted Setup**: Some schemes require time-consuming and complex trusted initialization.	(1) **Efficiency Gap**: Most schemes have not achieved an exponential gap between evaluation time and verification time; (2) **Mathematical Constraints**: Some schemes have specific prerequisites (e.g., requiring input curves to have unknown endomorphism rings); (3) **Engineering Optimization**: Practical performance of lattice-based schemes still needs significant improvement; (4) **Parallelism Dependency**: Some constructions require evaluators to have a certain degree of parallelism to exhibit advantages.
**Application Prospects**	Provides reliable delay for random beacons, consensus protocols, etc., in classical computing environments.	Aims to provide long-term secure delay primitives for key applications in the future quantum computing era (e.g., blockchain consensus, secure time stamping).

**Table 4:** Summary of typical attack methods and their targets.

Attack Method	Attack Target
Precomputation and TMTO Attack [40]	Sequentiality of PoSW and VDFs
Parallelization Attack (e.g., Pollard’s rho) [41]	Sequentiality of PoSW and VDFs
Shor’s Algorithm (Quantum Computing) [30]	Mathematical difficulty assumptions (e.g., integer factorization) of VDFs
Algebraic Shortcuts (e.g., using smooth numbers) [18]	Core mathematical operations (e.g., modular square roots) of VDFs
Low-Order Element Attack [42]	Uniqueness of VDF proofs
Structural Flaws in Mathematical Structures (e.g., supersingular isogeny cryptography) [42]	Uniqueness and reliability of VDF proofs
Hardware Acceleration [43]	Time-computation relationship of PoSW and VDFs
Side-Channel Attacks (e.g., timing, power analysis) [44,45]	Trapdoor key of tVDFs; secrets leaked by physical hardware
Grinding Attack [16]	Uniqueness of PoSW proofs; manipulation of protocol results
Selfish Mining [46]	Fairness and integrity of PoSW-based systems
DoS Attack [47]	Availability of verification nodes
Trusted Setup Compromise [48,49]	Security of public parameters and all VDF instances based on them

## Data Availability

The original contributions presented in this study are included in the article. Further inquiries can be directed to the corresponding authors.
